# Successful Dorsal Root Ganglion Stimulation for Chronic Pancreatitis: A Case Report

**DOI:** 10.7759/cureus.31852

**Published:** 2022-11-24

**Authors:** Tejas Shah, Ankur Khosla

**Affiliations:** 1 Physical Medicine and Rehabilitation, Hospital of the University of Pennsylvania, Philadelphia, USA; 2 Pain Management, AK Pain, Spine & Neuropathy, Woodlands, USA

**Keywords:** dorsal root ganglion, spinal cord stimulation (scs), neuromodulation, treatment of chronic pancreatitis, chronic pancreatitis

## Abstract

Chronic pancreatitis represents an inflammatory condition occurring from repetitive pancreatic inflammation episodes ultimately causing patients intractable pain alongside pancreatic insufficiency and as a result, reduced quality of life. In addition to alcohol and smoking cessation, patients with chronic pancreatitis are treated conservatively with anti-depressants, anti-convulsant and analgesic medications including paracetamol and celecoxib - with limited success. Alternative to surgical resection, patients can opt for endoscopic treatment options including sphincterotomy or removal of calculi which have shown limited success. Celiac plexus blocks have had positive outcomes as well, however, are not long-lasting and carry significant risks, such as gastroparesis or organ damage. Evidence has shown alterations in the peripheral and central nervous system which causes these patients to often experience dysfunctional and neuropathic pain. The advent of this knowledge has introduced neuromodulation into the field with successful cases of spinal cord stimulation treating the pain associated with chronic pancreatitis. Dorsal root ganglion stimulation is similarly based upon the gate theory of pain but with more precision as it strictly targets the dorsal root ganglion. There have been no cases reported in the literature of this form of stimulation in treating chronic pancreatitis. We present a case of a patient with chronic pancreatitis who obtained 80% abdominal pain relief for two years after undergoing dorsal root ganglion stimulation.

## Introduction

Chronic pancreatitis (CP) continues to be a plague on our healthcare system, affecting up to 75 per 100,000 patients a year around the world [1-3]. CP is a fibroinflammatory syndrome and is the culmination of repetitive pancreatic inflammation, ultimately leading to endocrine and exocrine insufficiency as well as fibrosis and intraductal calcifications-among other things [1,4]. Consequently, CP incurs significant healthcare costs, due in part to increased emergency room visits and high diagnostic and treatment costs [5,6]. Initial treatment involves lifestyle changes and medical management, including substantial opiate use [7]. Interventional options are limited for intractable abdominal pain (AP) secondary to CP and options involve stent placement and surgical consideration, with varied success [1,4]. Unfortunately, a consensus treatment option remains to be found; hence, neuromodulation has been introduced as another treatment option [1,3-6,8-11]. Spinal cord stimulation (SCS) has had remarkable success in providing pain relief for treatment-refractory AP secondary to CP [5,8,10-12]. Additionally, dorsal root ganglion (DRG) stimulation has also gained increased traction among providers given its more precise pain coverage; however, DRG stimulation has been utilized much less than its SCS counterpart, and, notably, there has not been a case utilizing DRG stimulation for CP-related AP [7,10,13]. Therefore, we present a patient with CP-related AP who experienced significant pain relief through DRG stimulation following a celiac plexus block (CPB).

This case was presented at the New York and New Jersey Pain Symposium on November 4, 2022.

## Case presentation

**Figure 1:**
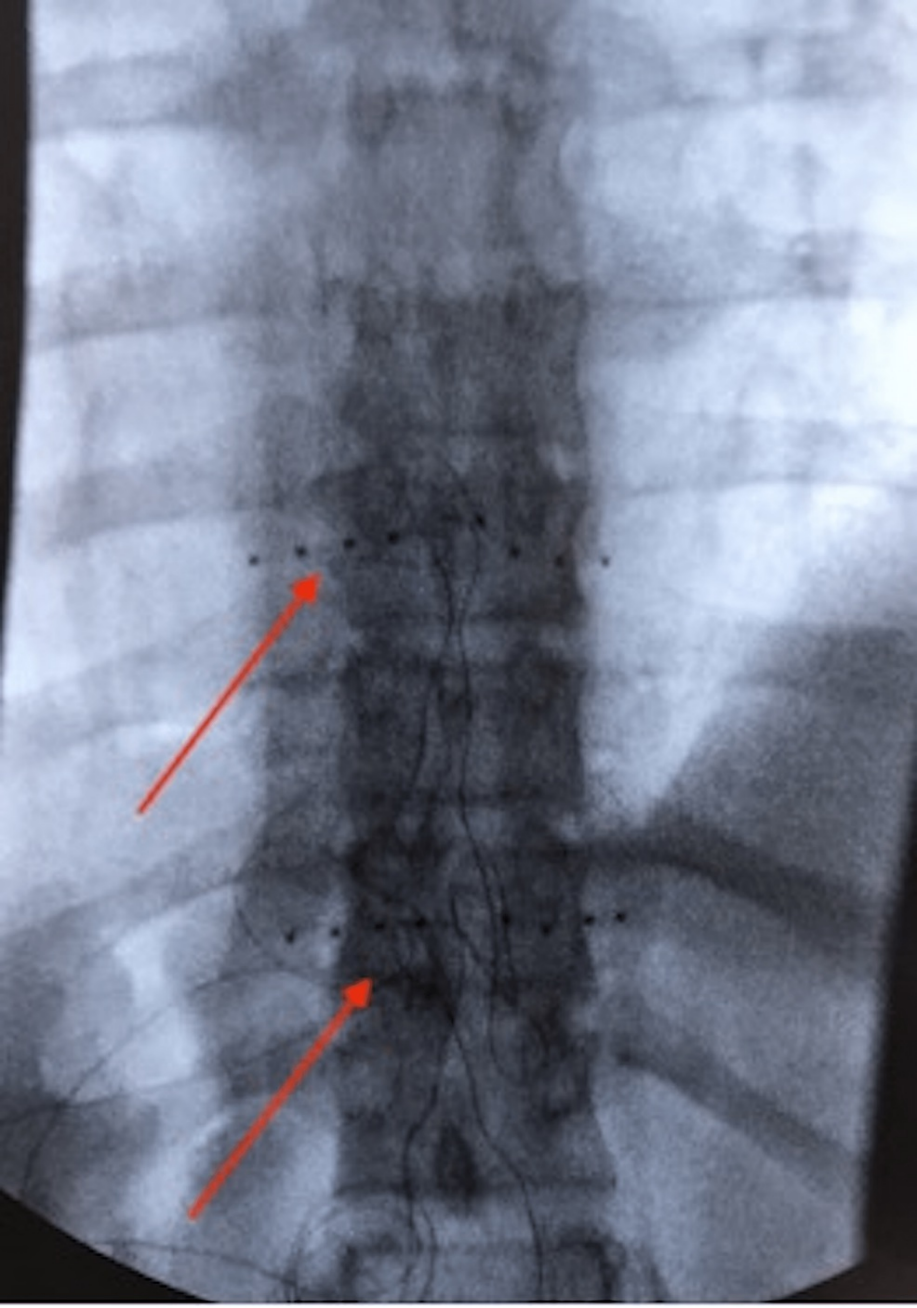
Intra-operative anterior-posterior fluoroscopy view of the thoracic spine with DRG leads (red arrows) at T8 and T10 vertebrae level.

A 34-year-old male with a past medical history of asthma, depression, insomnia, CP, and alcohol and tobacco use was referred to our clinic with a 6-month history of ongoing lower back and left-sided abdominal pain. The patient described the pain as “sharp, stabbing, and aching” and as located in the mid-to-left abdomen with associated radicular pain down his left leg more so than his right leg. Notably, the patient previously had taken codeine, amitriptyline, diclofenac sodium, ibuprofen, and meloxicam - without successful relief. During the time of the initial encounter, he was taking gabapentin and acetaminophen as needed. His initial physical exam revealed tenderness in the left upper quadrant of the abdomen without guarding or rebound tenderness. The abdomen was tympanic to percussion throughout. To confirm our suspicion of the pain’s etiology stemming from the abdomen, the patient was administered an ultrasound-guided CPB, with 80% resolution of pain from his baseline pain prior to the procedure. The patient, however, came back to the clinic two months later and reported his pain had returned. He reported the pain was similar to the level he had during the initial encounter. Due to the chronicity of pain as well as successful CPB, we recommended DRG stimulation to which the patient consented. The patient proceeded with a DRG trial at the bilateral T8 and T10 vertebrae levels with subsequent 85% pain resolution. As the patient experienced at least 50% pain relief, he was eligible to proceed with the permanent implant, to which he agreed. Temporary DRG stimulation leads were then taken out. A month later, the patient underwent a permanent DRG implant on the bilateral T8 and T10 levels (Figure 1). On his first post-operative visit, the patient reported 80% pain resolution. The patient was seen in the clinic one year later and noted continued 80% pain relief and stated he was able to resume physical activities, such as negotiating stairs, ambulating longer distances and working out at the gym. 

## Discussion

AP secondary to CP is a major burden on patients because it can persist for up to 10 years after the initial diagnosis [2]. AP is the fourth most common complaint among chronic pain patients and the most common presenting symptom of CP [4,6]. A variety of factors involved with CP play a vital role in its development; therefore, addressing these underlying factors will help in managing debilitating AP. A well-known risk factor, alcohol induces a toxic effect on pancreatic acinar cells via both oxidative and non-oxidative metabolites, and, not surprisingly, current or former alcoholic patients present relatively earlier with CP-related symptoms [2,4]. Alcohol is also commonly associated with exacerbations of acute pancreatitis, which may elucidate why a notable 35% of these patients will progress to CP within three to eight years of their first episode [4]. Smoking is another RF that has been correlated to faster progression from acute to CP [2,4]. These RFs were evident in our patient and may have played a role not only in the development of CP but also in the severity of the associated AP. Decreasing or eliminating such toxic habits would contribute to pain relief. Co-morbidities are common among chronic AP patients, who have been shown to have increased rates of depression, suicidal behavior and opiate use disorder [3,12]. The presence of psychological co-morbidities can exacerbate pain perception and increase the severity of CP-related AP in this patient population [5]. Finally, CP patients will suffer exocrine irregularities which contribute to gastrointestinal dysfunction and further exacerbate AP [2,7]. Therefore, pancreatic enzyme supplementation prevents the secretion of the pancreatic stimulation effects of cholecystokinin releasing factor which will spare the patient from additional pain and discomfort [7].

CP-related AP can arise via multiple avenues such as pancreatic inflammation, duct obstruction, and nerve damage [2,4,8]. As CP progresses, ongoing inflammation causes changes in the peripheral and central nervous systems, resulting in hypersensitivity of the pain response [2,8]. It has been proposed that the mechanical or chemical stimuli activate visceral nociceptive receptors, which convey pain signals via plexii to second-order neurons in the dorsal horns where pain signals ascent via the spino-thalamic and spino-reticular tracts [6]. Hence, CPB is done routinely to confirm an abdominal etiology for the pain-as in our case [11].

Despite the ongoing issues with the provision of adequate relief for chronic AP, neuromodulation - specifically electrical stimulation (ES) - has shown success as a potential therapeutic option. ES is theorized to work via the gate control theory, which involves stimulating large non-nociceptive Aβ fibers that inhibit, or “gate,” pain-carrying Aδ and C fibers [7]. In the case of DRG stimulation, this occurs at the dorsal horn of the spinal cord [7]. Both SCS and DRG stimulation have had notable success for various pain syndromes-such as chronic regional pain syndrome, peripheral neuropathies, and post-surgical or chronic lower back pain-but have had limited utilization in the GI realm [7]. In addition to relieving AP, neuromodulation directly affects the GI system, as it enhances vagal activity, improves visceral hypersensitivity and mucosal barrier function, and further reduces sympathetic tone to increase gastric emptying to improve GI discomfort [5,9,12]. A potential benefit of neuromodulation is the ability to help patients wean off opiate medications. Not only do opiates come with serious side effects, but its adverse effect of slowing GI motility worsens the patient’s underlying AP [12]. Both SCS and DRG stimulation are reversible, so every patient undergoes a trial period before moving on to implantation, an option that is not provided with abdominal surgeries [8]. Multiple case reports utilizing SCS for CP have noted improved pain scores and functional capacity alongside decreased opiate use [5,12]. Kapural et al. evaluated 30 patients with chronic AP from CP who underwent SCS and found that 80% of the study population reported greater than 50% pain reduction with an associated average visual analog scale (VAS), from 8 ± 1.6 centimeters (cm) reduced to 3.67 ± 2 cm [12]. Patients also reported a significant reduction in opiate use from 165 ± 120 morphine equivalents (ME) to 105 ± 101 ME per day [12]. The use of high frequency SCS is gaining preference among all SCS waveforms because of its increased therapeutic effect for axial back and radicular leg pain that is also paresthesia-free [5,14]. A recent study found that 23 of 24 patients who had a successful high-frequency SCS trial underwent permanent implantation [5]. Of these patients, 78.3% had at least 50% pain relief at 12 months, furthering the evidence in favor of SCS use [5]. With the success shown by SCS, we now turn our attention to another emerging waveform that applies ES to a more specific location in the back, the DRG [15].

The DRG is comprised of primary sensory neurons [7,10]. The high-density cluster of sensory information carries painful signals from the periphery to the brain via the spinal cord, where it is further processed [7,15]. SCS has had success in treating central neuropathic pain; however, it has been less reliable in focal or peripheral pain lesions, such as pain localized to the trunk, groin or foot [13]. With SCS, electrode leads project over the epidural space and often, non-painful areas are prone to unnecessary stimulation, and, consequently, patients complain of uncomfortable paresthesia [7,13]. In DRG stimulation, a more selective stimulation allows for coverage of focal painful lesions, especially those in a sub-dermatomal distribution [7]. Additionally, relatively less cerebrospinal fluid around the vicinity of the DRG correlates to lower electrical field dispersion and hence, a higher potent effect [7,15]. A common complication of SCS is lead migration [7]. Lead migration can cause a loss of therapeutic efficacy, mainly due to the increased distance created between SCS electrodes [7,15]. Although lead migration is possible with DRG stimulation, it is a lot less common because harnessing the lead around the DRG ensures that it is largely unbothered from sudden body movements and therefore less prone to movement [7]. Although much more limited in the literature, DRG stimulation has shown success in providing pain relief for AP [16,17]. Kloostermen et al. placed DRG stimulation leads at T11 after successful CPB for a patient with persistent AP, after which the patient reported 90% pain relief and was completely weaned off opiates [16]. DRG has also been used successfully for AP secondary to hereditary pancreatitis following cholecystectomy and total pancreatectomy with islet cell transplant [17]; however, there have not been any published cases discussing the successful utilization of DRG stimulation for CP-related AP.

## Conclusions

CP-related AP has unfortunately been difficult to quell. With an unclear consensus on standardized treatment, neuromodulation has shown limited success in significantly hampering the pain. SCS especially has had remarkable success for various types of AP, including that related to CP. DRG stimulation has shown an ability to capture areas of pain that are commonly missed by SCS. Although there are notable reports showcasing its utilization for AP, no cases regarding CP-related AP have been published. Our case showcasing significant pain relief for a patient with CP should pave the way for increased utilization of DRG stimulation in the CP patient population and potentially provide a more efficacious and minimally invasive therapeutic option.

## References

[1] Sharma V, Rana SS, Bhasin DK (2014). Medical management of pain in chronic pancreatitis. Trop Gastroenterol.

[2] Drewes AM, Bouwense SA, Campbell CM (2017). Guidelines for the understanding and management of pain in chronic pancreatitis. Pancreatology.

[3] Kim JK, Hong SH, Kim MH, Lee JK (2009). Spinal cord stimulation for intractable visceral pain due to chronic pancreatitis. J Korean Neurosurg Soc.

[4] Beyer G, Habtezion A, Werner J, Lerch MM, Mayerle J (2020). Chronic pancreatitis. Lancet.

[5] Kapural L, Gupta M, Paicius R (2020). Treatment of chronic abdominal pain with 10-kHz spinal cord stimulation: safety and efficacy results from a 12-month prospective, multicenter, feasibility study. Clin Transl Gastroenterol.

[6] Tiede JM, Ghazi SM, Lamer TJ, Obray JB (2006). The use of spinal cord stimulation in refractory abdominal visceral pain: case reports and literature review. Pain Pract.

[7] Liem L (2015). Stimulation of the dorsal root ganglion. Prog Neurol Surg.

[8] Vergani F, Boukas A, Mukerji N, Nanavati N, Nicholson C, Jenkins A (2014). Spinal cord stimulation for visceral pain related to chronic pancreatitis: report of 2 cases. World Neurosurg.

[9] Chen J (2022). Neuromodulation and neurostimulation for the treatment of functional gastrointestinal disorders. Gastroenterol Hepatol (N Y).

[10] Woodroffe RW, Pearson AC, Pearlman AM (2020). Spinal cord stimulation for visceral pain: present approaches and future strategies. Pain Med.

[11] Wie C, Ghanavatian S, Pew S (2022). Interventional treatment modalities for chronic abdominal and pelvic visceral pain. Curr Pain Headache Rep.

[12] Kannampalli P, Poli SM, Boléa C, Sengupta JN (2017). Analgesic effect of ADX71441, a positive allosteric modulator (PAM) of GABAB receptor in a rat model of bladder pain. Neuropharmacology.

[13] Potter ST, Welch S, Tata F, Probert S, Nagpal A (2022). Dorsal root ganglion stimulation. Phys Med Rehabil Clin N Am.

[14] Kapural L, Yu C, Doust MW (2016). Comparison of 10-kHz high-frequency and traditional low-frequency spinal cord stimulation for the treatment of chronic back and leg pain: 24-month results from a multicenter, randomized, controlled pivotal trial. Neurosurgery.

[15] Esposito MF, Malayil R, Hanes M, Deer T (2019). Unique characteristics of the dorsal root ganglion as a target for neuromodulation. Pain Med.

[16] Kloosterman JR, Yang A, van Helmond N, Chapman KB (2020). Dorsal root ganglion stimulation to treat persistent abdominal pain after bypass surgery. Pain Med.

[17] Justiz R, Smith N (2017). Thoracic DRG stimulation for chronic abdominal pain due to hereditary pancreatitis. Las Vegas: North American Neuromodulation Society.

